# The Mediator Role of Academic Resilience in the Relationship of Anxiety Sensitivity, Social and Adaptive Functioning, and School Refusal With School Attachment in High School Students

**DOI:** 10.3389/fpsyg.2020.00557

**Published:** 2020-04-21

**Authors:** İsmail Seçer, Sümeyye Ulaş

**Affiliations:** ^1^Counseling and Guidance, Faculty of Education, Ataturk University, Erzurum, Turkey; ^2^Counseling and Guidance, School of Health, Gümüşhane University, Gümüşhane, Turkey

**Keywords:** anxiety sensitivity, school refusal, academic resilience, mediation, social and adaptive functioning

## Abstract

School has an important function in providing the environment for young people to acquire many skills and knowledge required by contemporary life, but the problems of attachment to school and problematic attendance all over the world reveal an increasing statistic. It is thought that some negative processes such as anxiety sensitivity, social and adaptive functioning, and school refusal can affect this problem. On the other hand, it is considered that the academic resilience of young people has an important protective function in terms of these risk factors. For this purpose, the mediator role of academic resilience between anxiety sensitivity, social and adaptive functioning, and school refusal and school attachment were examined in a Turkish sample of 452 high school students. In the process of data collection, the school refusal assessment scale, social and adaptive functioning scale, and academic resilience scale were adapted and used in the Turkish culture. In the data analysis, the structural equation model was used to determine the direct and indirect predictive effects between the variables. The results of the study showed that academic resilience fully mediated the relationship between anxiety sensitivity and school attachment, whereas it partially mediated the relationship between social and adaptive functioning and school refusal and school attachment. Based on the results of the study, it was evaluated that high academic resilience has a strong protective function against the problems of negative school attachment and problematic school absenteeism among young people, and this finding was discussed within the context of literature.

## Introduction

School attachment and attendance are important for young people in terms of the environment they need for academic life; opportunities for the development of social competence and skills; and the ability to acquire professional skills, to solve problems, and to work together with others for a specific purpose (Tanner-Smith and Wilson, 2013; Kearney and Graczyk, 2014). Despite these advantages, the problems of school attendance and attachment among young people have become a worldwide problem. This situation, defined as problematic school absenteeism, is defined by Kearney (2008a) and Kearney and Graczyk (2014) as showing at least 25% absenteeism for a certain period (monthly, quarterly, etc.). This includes the part-time and full-time absenteeism of a student, as well as his/her planned behavior to be late for school in the morning (Peguero et al., 2011). Problematic absenteeism is a more widespread problem especially among young people and in low socioeconomic regions (Balfanz and Byrnes, 2012) and is mainly associated with school dropout (Rumberger, 2011). On the other hand, it has a pattern related to situations such as substance use, tendency to violence, suicidal tendency, risky sexual behaviors, and being involved in crime (Kearney, 2008b; Kearney and Graczyk, 2014; Aslan, 2018), and processes such as anxiety disorders, psychological adjustment problems, and developing antisocial behaviors (McShane et al., 2001; Knollmann et al., 2010; Rocque et al., 2017; Mazerolle et al., 2018). In this sense, it can be inferred from the results of these studies that problematic absenteeism is a problem area related to many of the personal, social, and academic characteristics of young people (Fornander and Kearney, 2019).

Researchers have found that the problem of school attendance is affected by the young people themselves, family, peers, and school environment (Burrus and Roberts, 2012; Ingul et al., 2012; Havik et al., 2014, [Bibr B23][Bibr B53]). Especially when the risks arising from family are considered, family functions including processes such as *domestic communication problems, role ambiguity, parental attitudes, and deterioration of family integrity* (Lagana, 2004) are thought to have an important place in this sense. It is thought that problematic domestic processes and deterioration in family functions may trigger school refusal, a problem that is thought to be closely related to the school attendance problem in young people by negatively affecting the process of turning to risky behaviors like school absenteeism (Jaycox and Repetti, 1993; Chen et al., 2017).

School refusal is defined as a phenomenon that includes severe symptoms like complete or partial absenteeism, chronically being late for school, developing deliberate behavior attempting to skip school in the morning, or accelerating the demand for future absence (Kearney and Bensaheb, 2006). School refusal is a problematic behavior that manifests with the child’s unwillingness to stay at school due to the strong negative emotions he/she feels at school and the desire not to come to school. It is also suggested that school refusal, which is considered an increasingly common condition in child psychiatry, should be considered as a child mental health problem (Kearney and Albano, 2004; Blumkin, 2016). However, studies on the diagnosis, evaluation, epidemiology, clinical features, follow-up, and treatment of school refusal are limited, and therefore, there is still controversy regarding the definition and evaluation of the concept of school refusal (Kearney and Bensaheb, 2006). Although school refusal was structured by Kearney and Silverman (1993), it was classified into four main categories by Heyne et al. (2019) as school refusal, truancy, school withdrawal, and school exclusion. However, the functional analytic approach proposed by Gonzálvez et al. (2019a) and especially by Kearney (2002) suggests that a combination of the two-dimensional processes of avoiding stressful situations and avoiding negative stimuli from school constitutes the main ground for school refusal. Studies have shown that school refusal, whose prevalence varies between 5 and 28% (Fornander and Kearney, 2019) among young people, is adversity that threatens the academic and normal lives of young people in the short and long term. Short-term outcomes include academic failure, being away from schoolwork, peer isolation, legal and financial difficulties, conflict with parents, and so on. Long-term outcomes include school dropout, feeling guilty, economic problems, difficulties in professional life and marriage, substance abuse, and adulthood psychological problems (Kearney and Bensaheb, 2006; Rocque et al., 2017; Mazerolle et al., 2018).

As explained above, school refusal is a common problem among young people, and although this problem is handled differently by professionals with different terminologies, it is often seen as an anxiety-based problem by psychologists (Last and Strauss, 1990; Kearney and Bensaheb, 2006; Richards and Hadwin, 2011; Kearney and Graczyk, 2014). Last and Strauss (1990) associate anxiety-based school refusal with separation anxiety disorder, which usually occurs due to incorrect attachment processes between mother and child. Phobias, another type of anxiety, appear to be an important factor in school refusal, and the concept of school phobia is used in some sources to replace school refusal (Hansen et al., 1998; Heyne et al., 2001; King and Bernstein, 2001; Egger et al., 2003; Kearney and Albano, 2004; Aaron and Cotler, 2009). Researchers found that anxiety-related disorders commonly associated with school refusal were separation anxiety (Hansen et al., 1998; Heyne et al., 2001; King and Bernstein, 2001; Egger et al., 2003; Kearney and Albano, 2004), generalized anxiety disorder (Heyne et al., 2001; Egger et al., 2003; Kearney and Albano, 2004), social anxiety disorder (Heyne and King, 2004; Kearney and Bates, 2005), mood disorders (Last and Strauss, 1990; Last et al., 1998; Egger et al., 2003), and social and specific phobia (Hansen et al., 1998; Heyne et al., 2001; King and Bernstein, 2001; Egger et al., 2003). However, although school refusal is considered as an anxiety-based disorder, it has been associated with anxiety sensitivity in recent years (Last and Strauss, 1990; King and Bernstein, 2001; Seçer, 2015; Aslan, 2018).

Anxiety sensitivity was explained by Petersan and Reiss (1992), with the expectation model of fear. Accordingly, it is considered that excessive fear and a tendency to avoid that anxiety-related symptom causing avoidance behavior in an individual may result in negativity due to any event or situation that causes fear (McNailly, 2002). Çakmak and Ayvaşık (2007), on the other hand, described it as “fear of fear” or “fear of anxiety” caused by the thought that the anxiety symptoms of the person would cause embarrassment and higher anxiety. Although anxiety sensitivity is clinically perceived as the same concept as expectation anxiety in panic disorder, it is a basic state of fear that exists in the structure of the person and shows continuity (Petersan and Reiss, 1992), and it has a function of reinforcing behaviors to avoid negative situations in the individual (McHugh and Otto, 2012), while expectation anxiety is defined as the anxiety that an individual experiences after panic attacks and that the individual will experience a panic attack again. Therefore, it is thought that anxiety sensitivity plays a role in the emergence and formation of school refusal, and in this way, it may trigger the problem of school attachment and problematic school absenteeism. Although there are still limited studies (Aslan, 2018) on the direct relationship between anxiety sensitivity and school refusal, researchers have found that anxiety sensitivity has a negative effect on the occurrence and maintenance of obsessive–compulsive disorder, panic attack, agoraphobia, depression, and other anxiety and mood disorders in young people (Cox et al., 1991; King and Bernstein, 2001; Grant et al., 2007; Mantar et al., 2011; Seçer, 2014a; Otto et al., 2016). Therefore, it is thought that anxiety sensitivity may increase the risk of avoidance reactions in young people against problematic situations. In this context, it is thought that anxiety sensitivity may be an important risk factor in terms of strengthening avoidance reactions in terms of coping with negative processes toward school (McHugh and Otto, 2012).

Social and adaptive functioning is another concept that is thought to be related to school attachment and problematic school absenteeism in young people (Gonzálvez et al., 2019a). Social and adaptive functioning is defined as a quality that includes cognitive, emotional, and linguistic processes related to a person’s social skills (Price et al., 2002; Crowe et al., 2011), and these processes have a significant impact on the individual’s personal, social and academic life (Gonzálvez et al., 2019b). As a matter of fact, researchers have demonstrated the effects of social and adaptive functioning on academic processes (Talwar et al., 2017; Vicent et al., 2017), negative peer and family relationships (Kandel and Davies, 1982), poor family relationships, and effective adaptation to school (Fernández-Zabala et al., 2016). The fact that social and adaptive functioning is related to school adaptation skills can be considered as an important protective variable, but it is thought that the relationship between school attachment, school refusal, skipping school, etc. has not been fully elucidated in the literature yet. The relationship between school refusal and social and adaptive functioning has been examined through four different school refusal profiles defined as *non–school refusers, school refusers by tangible reinforcements, and school refusers by negative reinforcements, and they found that non–school refusers* had a high level of functioning in all four structures (peer relationships, family relationships, and school performance and personal care) that constituted social and adaptive functioning, whereas *school refusers by mixed reinforcements* have low social and adaptive functioning, particularly in school performance and family and peer relationships. The results of this study suggest that there may be a strong relationship between school refusal and social and adaptive functioning and that social and adaptive functioning may have an important protective function for school refusal. Therefore, it can be considered that high social and adaptive functioning among young people is an important factor that shapes the problem of school attachment and problematic school attendance. It is thought that further experimental and empirical studies are needed to address relationship networks of these variables from an early age and to broaden our perspective in this direction (Gonzálvez et al., 2019b).

As explained above, problematic school absenteeism problems among young people are becoming a widespread problem throughout the world. In line with the information related to the literature, some qualifications such as anxiety sensitivity and school refusal deepen the problems of attachment to school and attendance among young people, whereas some skills such as social and adaptive functioning have a protective function. On the other hand, it is considered that the concept of academic resilience can have a regulatory function between the variables that have risk and protective characteristics and problematic school absenteeism during the occurrence and formation of problematic school absenteeism among young people. Psychological resilience is defined as overcoming the negative effects of risky situations that individuals are exposed to, successfully coping with traumatic experiences, and showing flexible and successful compliance despite the negative factors associated with these risks (Luthar et al., 2000; Masten and Powell, 2003, s. 1; Martin and Marsh, 2006). Bernard (1995) stated that social competence, problem-solving skills, and autonomy are related to being future-oriented and high future expectation, and Werner and Smith (1992) and Martin and Marsh (2006) stated that strong communication skills, effective time management, high sense of responsibility, being academically successful, being self-controlled, having high adaptation skills, and a positive self-perception are indicators of psychological resilience. In this context, academic resilience is defined as the tendency to show academic stability and success despite social and psychologically stressful and challenging life events (Alva, 1991; Benard, 1991; Wang et al., 1997; Perez et al., 2009). Students with high academic resilience are expected to show high levels of stability and success despite the presence and adverse effects of risky and stressful events (Alva, 1991; Martin and Marsh, 2006). In the literature, studies on the effects of academic resilience on school attachment and problematic school absenteeism are very limited. Ingul and Nordahl (2013) found that psychological resilience plays an important role in reducing school dropout among young people. Thus, resilience can play a key role in the emergence of problems such as school attachment, problematic school absenteeism, and school dropout. In this context, considering the variables mentioned above, it is thought that high anxiety sensitivity and school refusal behavior among the young pose a significant risk on school attachment and problematic absenteeism (Aslan, 2018), but academic resilience can reduce this risk. It is considered that through academic resilience, school attachment processes will be affected positively by social and adaptive functioning, which has positive effects on school attachment and the reduction of problematic school absenteeism. In this sense, it is thought that academic resilience may have a mediatory function among these variables and affect school attachment and problematic school absenteeism in young people.

### The Current Study

The purpose of this study is to examine the mediating role of academic resilience between school attachment and anxiety sensitivity, social and adaptive functioning, and school refusal among young people. For this purpose, the research process is structured around the following questions: 1. Do anxiety sensitivity, social and adaptive functioning, and school refusal predict school attachment? 2. Does academic resilience play a mediating role in the relationship of anxiety sensitivity, school refusal, and social and adaptive functioning with school attachment in young people? Determining the possible mediator role of academic resilience between school attachment and anxiety sensitivity, social and adaptive functioning, and school refusal is considered to contribute to broadening our perspective and shaping intervention and action plans for reducing problematic school attendance problems among young people. Although there is a good amount of fund of knowledge related to school refusal with the scientific studies conducted on problematic school attendance specific to Turkey, it is not possible to say that school refusal, social and adaptive functioning, academic resilience, etc. are not yet sufficiently addressed with problematic school attendance in Turkey. It is believed that this is because an adequate level of fund of knowledge hasn’t been formed sufficiently to expand the perspectives of field experts and field workers, and therefore, preventive and rehabilitative studies are limited. Thus, it is thought that the results obtained from this study will deepen the perspectives on the nature of the problematic school attendance among young people in Turkey. In this context, answers to the following questions were sought in the research process.

1 Are anxiety sensitivity, social and adaptive functioning, and school refusal significant predictors of school attachment in young people?
2 Is academic resilience a significant predictor of school attachment?
3 Does academic resilience play a role in the predictive relationship of anxiety sensitivity, social and adaptive functioning, and school refusal with school attachment?

## Materials and Methods

### Participants

The participants of the study consisted of 452 high school students (with an average of age of 15.13, *sd* = 1.64) aged between 13 and 18. Of the participants, 47.8% were males and 52.2% were females. A two-stage process was followed in the process of identifying the participants. In the first stage, high schools were grouped according to the cluster sampling method, and the schools to be sampled by random sampling were determined. In the process of identifying the students to be included in the data collection process from the selected schools, the convenience sampling method was applied. In this process, teachers’ and school psychologists’ opinions were taken into consideration in order to identify the participants. Therefore, the guidance of school counselors was particularly used in order to include children who attend school regularly as well as children with problematic school absenteeism. The participants consisted of young people, 31% of whom did not have any problematic attendance in the last term, 29% of whom had between 1 and 3 days of absenteeism, 24% of whom had between 4 and 6 days of absenteeism, 10% of whom had between 7 and 10 days, and 5% of whom had 11 days. In addition, when the distribution of participants in terms of school refusal profiles is examined, 61% of them are in the *non–school refusers group*, 21% are in the *school refusers by mixed reinforcements group*, 10% are in the *school refusers by tangible reinforcements group*, and 8% are in the *school refusers by negative reinforcements group*.

## Measures

### School Refusal Assessment Scale

It was developed to evaluate school refusal behavior in children and adolescents by Christopher and Silverman (1993) and revised by Heyne et al. (2017). The scale is a Likert-type scale consisting of 24 items and four sub-dimensions. In the revision process, the scale was tested on 24 items with the addition of new items by removing some items in the first version, and it was observed that it included 22 items and four sub-dimensions. The pre-revision form of the scale was adapted to Turkish culture by Seçer (2015), and it was determined that the form consisting of a total of 19 items and four sub-dimensions was compatible with the Turkish culture. The psychometric properties of the revised form were also examined with 485 children and adolescents aged 10–18 years. In the adaptation process, Heyne et al. (2017) tested the two items that were found to be not a good fit, and the four-factor structure of the scale consisting of 24 items was found to be a good fit in the Turkish culture (χ^2^/*sd* = 2.21, RMSEA = 0.061, NFI = 0.97, CFI = 0.98, GFI = 0.94). The Cronbach alpha value for the reliability analysis of the scale was found to be 0.85 for the scale total, and 0.87, 0.85, 0.83, and 0.84 for the sub-dimensions, respectively. As a result of the analysis of the factor structure and reliability of the scale in the adaptation process, it was evaluated that the psychometric properties of the scale were sufficient (Seçer, 2015). The sub-dimensions of the scale are avoidance of negative situations related to school, having difficulty in engaging socially, resisting to leave parents, and being interested in out-of-school activities. The scale is scored as 1 (never) to 4 (always), and the scores on the scale range from 24 to 96. High scores obtained from the sub-dimensions and the total of the scale indicate a high level of school refusal.

#### Anxiety Sensitivity Index

It is a Likert-type scale developed by Silverman et al. (1991) and adapted to Turkish culture by Seçer and Gülbahçe (2013). The scale consists of 15 items and three sub-dimensions, physical, psychological, and social. The adaptation process of the scale was carried out with children and adolescents aged 12–18 years. In this research process, the validity of the model fit of the scale was re-examined with confirmatory factor analysis, and the fit indices (χ^2^/*sd* = 1.06, RMSEA = 0.023, NFI = 0.9, CFI = 0.99, GFI = 0.92) were determined to be good. The Cronbach alpha of the scale was 0.82, 0.91, and 0.90 for the sub-dimensions, respectively. The scale is scored as 1 (never) to 5 (generally), and the scores that can be obtained from the scale vary between 15 and 75. High scores on the subscales and the total of the scale indicate a high level of anxiety sensitivity.

#### Academic Resilience Scale

It is a Likert-type scale developed by Cassidy (2016) to measure processes related to academic resilience and includes four sub-dimensions. Although the original scale form was developed for university students, the psychometric properties of the high school population of 327 people were also examined during the adaptation process to Turkish culture (Ulaş and Seçer, 2020). Findings from the high school population indicated that the scale’s 22 items and three sub-dimensions were well adapted to Turkish culture (χ^2^/*sd* = 2.16, RMSEA = 0.062, NFI = 0.98, CFI = 0.98, GFI = 0.96). The Cronbach alpha for the sub-dimensions was 0.82, 0.79, and 0.82, respectively. The sub-dimensions of the scale are perseverance, reflecting and adaptive help-seeking, negative effect, and emotional response. The scale is scored as 1 (never) to 4 (always), and the scores on the scale range from 22 to 88. High scores obtained from the sub-dimensions and the total of the scale indicate a high level of academic resilience among the youth.

#### Social and Adaptive Functioning Scale

It is a self-report measure developed by Price et al. (2002) to examine social and adaptive functioning in children and the young. The scale was adapted to Turkish culture as a part of this research process, and its psychometric properties were examined. After conducting linguistic equivalent studies and pilot applications, the psychometric properties of the scale were examined. In this context, the construct validity of the scale was examined with 341 high school students between the ages of 14 and 18. The results obtained from the confirmatory factor analysis showed that the scale form consisting of 20 items and four sub-dimensions was well adapted in Turkish culture (χ^2^/*sd* = 2.25, RMSEA = 0.057, NFI = 0.98, CFI = 0.98, GFI = 0.96). The Cronbach alpha for the sub-dimensions was 0.83, 0.81, 0.79, and 0.84 for the sub-dimensions, respectively. The sub-dimensions of the scale were family relationships, peer relationships, home duties, and school performance. The scale is scored as 1 (never) to 4 (always), with scores ranging from 20 to 80. High scores obtained from the sub-dimensions and the total of the scale indicate a high level of social and adaptive functioning in young people.

#### School Attachment Scale

It is a self-report scale developed by Hill and Werner (2006) in order to evaluate the level of attachment of children and adolescents to school and adapted to Turkish culture by Savi (2011). In the adaptation process of the scale, exploratory factor analysis was performed, and it was observed that the scale, which consisted of 15 items and three sub-dimensions in its original form, had a good fit with 13 items and three sub-dimensions in Turkish culture. In this research process, the psychometric properties of the scale were re-examined, and it was determined that the scale maintained the model fit (χ^2^/*sd* = 2.96, RMSEA = 0.071, NFI = 0.95, CFI = 0.96, GFI = 0.94). The Cronbach alpha values were 0.78 for attachment to school, 0.81 for attachment to teacher, and 0.83 for attachment to friend. The scale is scored as 1 (never) to 4 (always), with scores ranging from 13 to 52. High scores obtained from the sub-dimensions and the total of the scale indicate a high level of attachment to school among young people.

## Procedure and Data Analyses

In the first stage of the study, two different procedures were performed. In the first-procedure stage, research permission was obtained from Atatürk University Educational Sciences Ethics Committee, and in the second-procedure stage, necessary permissions were obtained from local administrators for conducting the research. Parents’ approvals were gotten through the school administrations after the permissions had been granted, and measurement tools were applied to the students who wanted to participate only voluntarily under the guidance of the school counselor. The data collection process took ~15 days, and the application period of the measurement tools took ~20 min. Data collection was carried out by two researchers with expertise in the field of psychology and psychological counseling. Optical forms were used in the data collection process, and the OMR REMARK survey program was used to transfer the collected data to the computer environment. For the data transferred to the computer environment, missing data analysis was first performed by SPSS 21 software, and the scales containing 5% loss data were removed from the data set as suggested (Bell et al., 2009; Graham, 2009). In this context, data belonging to 11 people were excluded from the scale form. In the second stage, skewness, kurtosis, and Mahalanobis and Cook’s calculations were made for extreme value analysis, and it was decided to extract the data belonging to nine people. In the third stage, the normality values were examined by LISREL9 software, and it was found that the data set showed normal and homogeneous distribution when the transformation process was applied.

After the parametric conditions had been fulfilled, the confirmatory measurement model and structural equation models were tested in order to seek answers to the research questions. Three different models were tested in the structural equation model. In Model 1, it was tested whether anxiety sensitivity, school refusal, and social and adaptive functioning directly predicted school attachment. In Model 2, academic resilience was included in the model with anxiety sensitivity, and it was tested whether school refusal and social and adaptive functioning predicted school attachment both directly and through academic resilience. In Model 3, the full mediating role of academic resilience among these variables was tested. Schumacher and Lomax (2004) and Tabachnick and Fidell (2013) suggest that the fit indices in structural equation modeling should be ≥0.90 for acceptable fit and ≥0.95 for perfect fit for, TLI (Tucker-Lewis Index), CFI (Comparative Fit Index), NFI (Normed Fit Index), NNFI (Non-Normed Fit Index), and IFI (Incremental Fit İndex); ≥0.85 for acceptable fit and ≥0.90 for perfect fit for GFI (Goodness-of-Fit Index) and AGFI (Adjusted Goodness-Of-Fit Index); and ≤0.08 for acceptable fit and ≤0.50 for perfect fit for RMR (Root Mean Square Residual), REMSEA (Root Mean Square Error of Approximation), and SRMR (Standardized Root Mean Square Residual). A two-stage process was followed in the data analysis process. In the first stage, the confirmatory measurement model was applied for the fit of the hypothesized models. Five different implicit variables (anxiety sensitivity, school refusal, social and adaptive functioning, academic resilience, and school attachment) and 17 observed variables represented by these implicit variables were used in the confirmatory measurement model. The verification of measurement models is an important prerequisite for testing structural equation models (Şimşek, 2007, s. 117). The indices of fit obtained from the measurement model are as follows:[χ^2^ (109) = 211.67/*sd* = 1.94; CFI = 0.96; GFI = 0.95; SRMR = 0.06; RMSEA = 0.06]. These show that all implicit variables fit well with the indicator variables they represent and other implicit variables (Tabachnick and Fidell, 2013). The models created for the purpose of the research were tested with a two-stage process. In the first stage, the direct predictive effects of anxiety sensitivity, school refusal, and social and adaptive functioning on school attachment were tested. In the second stage, the mediation role of academic resilience among these variables was examined, and indirect effects were determined.

## Results

**FIGURE 1:**
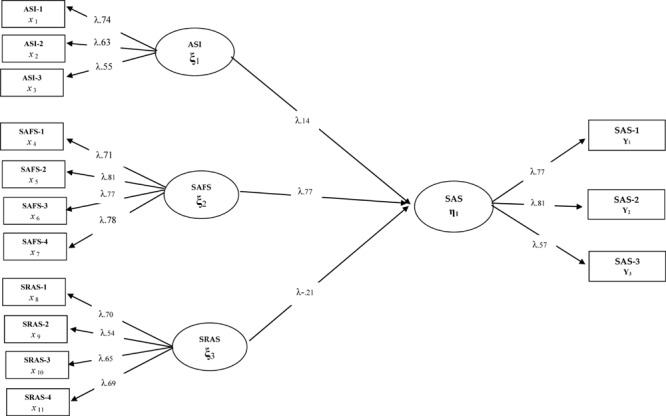
Standardized SEM results for Model 1.

After the validation of the measurement model, three different models that were formed for the purpose of the research were tested respectively. In this context, Model 1 tested the direct predictive effect of anxiety sensitivity, school refusal, and social and adaptive functioning on school attachment. In Model 1, anxiety sensitivity and social and adaptive functioning are expected to predict school attachment positively, and school refusal predicts school attachment negatively. The obtained findings related to Model 1 are presented in Figure 1.

When the fit indices [χ^2^(71) = 187.34/*sd* = 2.43; CFI = 0.94; GFI = 0.92; RMSEA = 0.077] of the model tested in Figure 1 are considered, it can be said that all the implicit variables in Model 1 have a significant relationship with the observed variables they represent (*p* < 0.01). Model 1 shows that three implicit variables explaining school attachment fit well, anxiety sensitivity and social and adaptive functioning predict school attachment positively, and school refusal predicts school attachment negatively as expected (β = 0.77, *p* < 0.01, β = -0.21, *p* < 0.01, β = 0.14, *p* < 0.01). When the findings and explanation coefficients are taken into consideration, it is understood that social and adaptive functioning has a strong effect on school attachment (59%), followed by school refusal and anxiety sensitivity, respectively.

**FIGURE 2:**
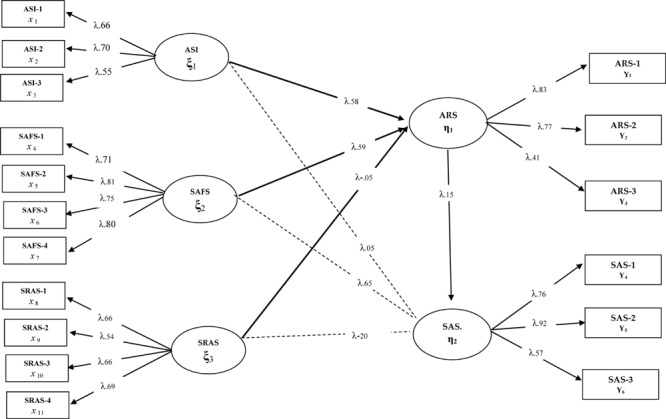
Standardized SEM results for Model 2.

After verification of the hypothesis in Model 1, the second stage of mediation relationships should be applied. At this stage, the mediating effect of the model is included, and the parameters related to the direct and indirect relationship processes between the predicting variables and the predicted variable are examined. In this context, academic resilience was included in the model designed in Model 1 between anxiety sensitivity, social and adaptive functioning, and school refusal and school attachment, and it was tested as Model 2. The findings related to Model 2 are presented in Figure 2.

**FIGURE 3:**
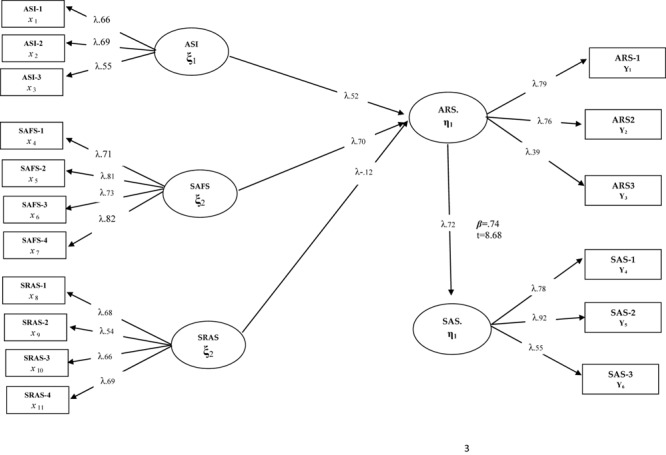
Standardized SEM results for Model 3.

Considering the findings of Figure 2, a significant change was observed in the parameters reached in Model 1 after the academic resilience variable had been included in the model. Considering the model fit indices, it is understood that the fit indices of Model 2 are not sufficient [χ^2^(94) = 565.20/*sd* = 6.01; CFI = 0.92; GFI = 0.089; SRMR = 0.10; RMSEA = 0.10]. On the other hand, while anxiety sensitivity had a significant effect on school attachment (β = 0.14, *p* < 0.01) in Model 1 when Figure 2 was examined, this significant relationship disappeared after including the academic resilience variable (β = 0.05, *p* > 0.01) in Model 2. School refusal had a significant effect on school attachment (β = 0.31, *p* < 0.01) in Model 1, but it decreased (β = -0.20, *p* > 0.01) after including the academic resilience variable to the model. In terms of social and adaptive functioning, it is seen that the correlation coefficient in Model 1 (β = 0.77, *p* < 0.01) shows a significant decrease with the addition of the academic resilience variable (β = 0.65, *p* < 0.01). With the addition of the academic resilience variable to the model, observing a significant change in the relationship coefficients between the variables can be considered as a strong sign that mediation relationships may exist. In addition, when Figure 2 is examined, it is seen that the predictive effect of academic resilience on school attachment is not significant if there are direct and indirect paths between the variables (β = 0.15, *p* < 0.01). For this reason, the full mediating role of the academic resilience variable was tested by removing the direct paths from anxiety sensitivity, school refusal, and social and adaptive functioning variables to school attachment. This model, called Model 3, deals with the full mediation relationships between variables. The structural model dealing with the full mediation relationship is presented in Figure 3.

The indices and parameters of Model 3 testing the full mediating role of academic resilience [χ^2^(97) = 156.79/*sd* = 1.61; CFI = 0.97; GFI = 0.96; SRMR = 0.053; RMSEA = 0.054] show that the mediation of the tested model and academic resilience is verified. When Figure 3 is examined, it can be seen that anxiety sensitivity (β = 0.62, *p* < 0.01), social and adaptive functioning (β = 0.70, *p* < 0.01), and school refusal (β = 0.12, *p* < 0.01) predicted school attachment through academic resilience. Considering the Model 2 parameters, it is understood that there are significant improvements in the statistical values after removing the paths showing low or insignificant predictions from the model. Therefore, both the good fit of the hypothesized model and the observation of a significant change in the path coefficients between the variables were considered as indicators of the mediating role of academic resilience. In addition, the predictive effect of academic resilience on school attachment was determined as β = 0.72, *p* < 0.01. Compared to Model 2, it is thought that there is a significant increase in the predictive coefficient of academic resilience on school attachment (β = 0.15, *p* < 0.01) and that these values are obtained by subtracting the low or insignificant relationship paths in Model 2 from the model.

## Discussion

### The Relationship Process of Anxiety Sensitivity, School Refusal, and Social and Adaptive Functioning With School Attachment and the Mediator Role of Academic Resilience

According to the results of the study, the predictive effect of variables that have a predictive effect on school attachment in youth can be discussed in two ways. The first is direct effects, and the second is indirect effects. The effects of anxiety sensitivity, social and adaptive functioning, and school refusal on school attachment can be discussed as direct effects. The predictive effect of anxiety sensitivity, school refusal, and social and adaptive functioning on school attachment can be discussed through the academic resilience variable as indirect effects.

The results of the study showed that anxiety sensitivity had a positive effect on school attachment. Anxiety sensitivity is explained by the “expectancy model of fear” in the relevant literature (Reıss and McNally, 1985; Çakmak and Ayvaşık, 2007). In other words, the individual has an intense expectation that negative situations will emerge, and he/she reacts to avoid and has a feeling of fear toward certain negativities that may occur in school. In this respect, it is thought that anxiety sensitivity is likely to turn into a pressure tool on problematic school absenteeism processes. Based on the results of the research that reveals the relationship between anxiety sensitivity and psychological problems such as mood disorders, depression, agoraphobia, and OCD (Obsessive Compulsive Disorders) (Cox et al., 1991; King and Bernstein, 2001; Grant et al., 2007; Mantar et al., 2010; Seçer, 2014a), it can be considered that a high anxiety sensitivity level will have a negative effect on school attendance in young people. In addition, although anxiety sensitivity had a low predictive effect on school attachment in Model 1, this effect disappeared in Model 2, in which academic resilience was included in the analysis, which means anxiety sensitivity does not have a direct effect on school attachment and strongly influences school attachment through the academic resilience variable.

Including the academic resilience variable in Model 2, the direct predictive effect of anxiety sensitivity on school attachment disappeared, indicating the mediator role of academic resilience and that type II error was prevented. Academic resilience is seen as a dimension of psychological resilience, and it is defined as showing academic stability and success despite the psychological and social stressors encountered in school-related processes and challenging academic processes (Wang et al., 1994; Perez et al., 2009). In this respect, it can be said that academic resilience is an important protective feature in terms of school attachment and overcoming problematic school absenteeism problems. In the literature, there are very limited study findings that address the effect of academic resilience on processes like school attachment. The results obtained from these studies show that academic resilience is an important factor in preventing school dropout problems in young people (Ingul and Nordahl, 2013). The results obtained from Model 2, which tested the full mediation of academic resilience, show that anxiety sensitivity predicts academic resilience and academic resilience predicts school attachment in a positive and powerful way. Based on these results, it is considered that contrary to what is believed, anxiety sensitivity does not have a completely negative quality and is a factor that reinforces academic resilience in young people and positively affects school attachment processes in young people. Nonetheless, it should be taken into consideration that the fact that there are very limited research findings significantly limited our perspective on the interpretation of the results obtained from the research.

The second variable whose direct and indirect effects were examined on school attachment was social and adaptive functioning. Social and adaptive functioning is defined as a quality that includes cognitive, emotional, and linguistic processes related to an individual’s social skills (Price et al., 2002; Crowe et al., 2011). The results of the study showed that social and adaptive functioning positively and strongly predicted school attachment in young people in Model 1, but there was a significant decrease in the predictive coefficient with the inclusion of academic resilience in Model 2. The obtained results indicate that social and adaptive functioning predicts school attachment both directly and indirectly through academic resilience. Therefore, the continuation of the direct impact after the academic resilience variable was included in the model indicates a partial mediation relationship. Nevertheless, although studies on the impact of social and adaptive functioning on school attachment processes are limited (Talwar et al., 2017; Vicent et al., 2017; Gonzálvez et al., 2019b), it is seen that they support the findings obtained from this study. Therefore, it is thought that social and adaptive functioning has a very strong protective feature in terms of overcoming the problems of school attendance and problematic school absenteeism among young people, and high academic resilience reinforces this effect. In other words, a high level of social and adaptive functioning and high academic resilience are considered to be a powerful tool in ensuring positive school processes in young people. As in anxiety sensitivity, the fact that there are a limited number of studies in the literature for social and adaptive functioning can be considered as a factor limiting the perspective in this direction and weakening the interpretations. Therefore, it is clear that more research findings are needed in this direction.

The third variable whose direct and indirect effect on school attachment was examined in the study is school refusal. School refusal is an anxiety-based problem that is related to complete or partial absenteeism, chronically being late for school, developing deliberate behavior attempting to skip school in the morning, or accelerating the demand for future absence (Kearney and Bensaheb, 2006). The findings of the study show that school refusal, which has become a widespread problem among young people, negatively and directly predicts school attachment. This finding is consistent with the literature, and school refusal is a problem that triggers problematic school absenteeism problems among young people. However, it is thought that academic resilience plays an important role in limiting the negative effect of school refusal on school attachment. The findings of the study show that school refusal predicts academic resilience negatively. Considering the positive role of academic resilience in school attachment, it is thought that a high level of academic resilience may serve as a protective function in terms of possible school refusal behavior in young people, which provides a basis for school attachment and problematic school absenteeism problems. Based on studies that reveal the relationship between separation anxiety (Hansen et al., 1998; Heyne et al., 2001; King and Bernstein, 2001; Egger et al., 2003; Kearney and Albano, 2004), generalized anxiety disorder (Heyne et al., 2001; Egger et al., 2003; Kearney and Albano, 2004), social anxiety disorder (Heyne and King, 2004; Kearney and Bates, 2005), and mood disorders in children who refuse school, it can be said that school refusal is likely to turn into a pressure tool on school attachment and attendance problems due to the close relationship with psychological problems among young people. Therefore, it is thought that academic resilience plays a protective role in reducing or even preventing the negative effects of school refusal and related psychological problems on school attachment and attendance processes of young people. Although limited research findings in this field limit our point of view, it is considered that high academic resilience will have a protective function against problematic school attendance problems that may arise due to school refusal in young people and positively affect school attachment.

## Limitations and Future Research

The findings of this study should be evaluated in the context of its limitations. Firstly, the relational and cross-sectional nature of the study and the fact that the sampling process relies heavily on convenience sampling have an important limitation in terms of establishing cause–effect relationships. In addition, measuring the qualifications of young people based solely on self-reporting is an important limitation. Therefore, the choice of mixed research approaches, including the views of parents, teachers, etc., through triangulation, may offer a broader perspective. In addition, conducting the research only with high school students is another limitation. Therefore, it may broaden our perspective to diversify similar research, including other teaching levels and age groups. Another limitation is that the research findings were conducted only with children from a Turkish sample. It is considered that conducting similar research in different cultures and countries will make significant contributions to the literature in order to understand the cultural aspects of problematic school absenteeism problems among young people.

## Implications

The results of the research are considered to have significant effects for both relevant researchers and school counselors and school psychologists. It is considered that determining the protective role of academic resilience in terms of school attachment and overcoming problematic school absenteeism problems in young people will shed light on the preventive and intervention practices of school professionals and broaden their perspectives. For the researchers, it is expected that this will provide important impacts in terms of testing holistic and causal models for understanding the problems of school attachment and problematic school absenteeism and revealing a theoretical process for developing applications especially for strengthening academic resilience.

## Data Availability Statement

All datasets generated for this study are included in the article/supplementary material.

## Ethics Statement

The studies involving human participants were reviewed and approved by Atatürk University Educational Sciences Ethics Committee. The patients/participants’ guardian provided their written informed consent to participate in this study.

## Author ContribuTions

As a result of the literature review of the relevant field, İS and SU acted jointly in the process of revealing the research idea. After determining the subject of the research, İS and SU took an active role in completing the research procedures. İS and SU conducted the research process, like research permits, ethics committee approvals, and interviews with schools to collect data. The data collection process was a process that İS and SU carried out together. The transfer of the collected data to the computer environment and the examination of their suitability and parametric test conditions for analysis were done by İS. Data analysis and reporting processes were completed by SU. In the writing process of the study, the *Introduction* and *Discussion* parts were completed by İS significantly, and SU contributed to this process. The methods and findings sections were prepared for publication by SU and contributed by İS. During the publication of the article, the feedback from the editors and the referees was organized together by İS and SU.

## Conflict of Interest

The authors declare that the research was conducted in the absence of any commercial or financial relationships that could be construed as a potential conflict of interest.
